# Differential Glycosylation Patterns in Parkinson’s Disease: Emphasis on Male-Specific Changes Identified via HILIC-LC-MS

**DOI:** 10.3390/ijms27010552

**Published:** 2026-01-05

**Authors:** Béla Demeter, Adriána Kutás, Béla Viskolcz, Csaba Oláh, Edina Petercsák, Attila Garami, Csaba Váradi

**Affiliations:** 1 Institute of Chemistry, Faculty of Materials Science and Chemical Engineering, University of Miskolc, 3515 Miskolc, Hungary; demeter.bela@gmail.com (B.D.); kutas.adriana@student.uni-miskolc.hu (A.K.); bela.viskolcz@uni-miskolc.hu (B.V.); 2 Borsod-Abaúj-Zemplén County Center Hospital and University Teaching Hospital, Department of Neurosurgery, 3526 Miskolc, Hungary; olahcs@gmail.com (C.O.);; 3 Institute of Energy, Ceramic and Polymer Technology, University of Miskolc, 3515 Miskolc, Hungary

**Keywords:** Parkinson’s disease, glycosylation, liquid chromatography

## Abstract

Parkinson’s disease (PD) is a progressive neurodegenerative disorder primarily characterized by the degeneration of dopaminergic neurons, leading to significant motor and non-motor symptoms. This study investigates glycosylation patterns with a significant emphasis on male Parkinson’s Disease (PD) patients, revealing unique alterations distinguishing PD from healthy states, utilizing high-performance liquid chromatography coupled with mass spectrometry (HILIC-LC-MS). Findings reveal significantly altered serum N-glycosylation profiles between male and female patients, with increased levels of high-mannose glycans and reduced mono-sialylated glycans in male patients. ROC curve analysis indicates that these glycan changes are the most important features for distinguishing PD from healthy states, with AUC values of 0.71 for M5 and 0.85 for M6. This study underscores the critical role of glycosylation in the pathophysiology of Parkinson’s disease and highlights its potential in early detection and monitoring of disease progression.

## 1. Introduction

Parkinson’s disease (PD) is described as a progressive neurodegenerative disorder that primarily affects movement control [1]. It is characterized by the degeneration of dopaminergic neurons in the substantia nigra, which is a crucial area of the brain that plays a significant role in coordinating movement [2]. The main symptoms of PD include tremors, rigidity, bradykinesia (slowness of movement), and postural instability [3]. As the disease progresses, non-motor symptoms such as cognitive decline, depression, and autonomic dysfunction may also be experienced by patients [4]. These symptoms significantly impact the quality of life, leading to increased dependency and reduced social interactions among affected individuals [5]. Glycosylation is recognized as a post-translational modification involving the attachment of carbohydrate moieties to proteins and lipids [6]. This process is critical for the proper functioning of many biological systems [7]. Glycans are known to influence protein folding, stability, and activity, and play essential roles in cell–cell communication, immune response, and the modulation of protein interactions [8]. Two main types of glycosylation are identified: N-linked and O-linked glycosylation, which differ in the types of linkages and the amino acid residues involved [9]. The intricate nature of glycosylation highlights its potential as a regulatory mechanism in various cellular processes, including those affected in neurodegenerative diseases [10]. In the context of neurodegenerative diseases, including Parkinson’s disease, glycosylation has emerged as a significant area of research [11]. Alterations in glycosylation patterns have been observed in the brains and bodily fluids of PD patients, suggesting that these changes may be implicated in disease onset and progression [12]. Recent studies have indicated that specific glycan structures can modulate the inflammatory response, which is particularly relevant given the emerging role of neuroinflammation in the progression of PD [13]. Biomarkers derived from altered glycosylation patterns could enhance the accuracy of PD diagnosis, enabling earlier therapeutic interventions [14]. Recent advancements in analytical chemistry have enhanced our understanding of glycosylation’s role in diseases, including Alzheimer’s disease (AD). A study demonstrated the potential of glycan profiling to identify biomarkers associated with AD. Our research builds on this foundation by examining glycosylation patterns in Parkinson’s disease (PD) patients, focusing on differences by gender and disease status, to explore their diagnostic potential [15]. Moreover, it has been suggested that therapeutic strategies targeting glycosylation pathways could have the potential to modulate disease processes and improve outcomes for patients with PD [16]. Glycosylation, as a critical post-translational modification, is viewed as playing a vital role in numerous biological processes and may be intricately linked to the mechanisms underlying PD [6]. Understanding the interplay between glycosylation and Parkinson’s disease could pave the way for novel diagnostic and therapeutic approaches, ultimately enhancing the quality of life for patients battling this challenging condition [17]. As research continues to elucidate the complexities of glycosylation in neurodegeneration, hope is offered for the development of innovative strategies to combat Parkinson’s disease and improve patient outcomes [18].

The aim of this study is to investigate the serum N-glycosylation profiles in male patients diagnosed with Parkinson’s disease (PD) compared to healthy controls. Distinct glycan alterations that may help differentiate PD from healthy states are sought. By focusing on these sex-specific differences, a deeper understanding of the underlying biological mechanisms of PD is aimed to be achieved, informing personalized diagnostic and therapeutic strategies.

## 2. Results

### 2.1. Serum N-glycosylation-Based Differences Between Parkinson’s Disease and Healthy Control Patients

In this study, the serum N-glycosylation profiles of patients diagnosed with Parkinson’s disease (PD) were analyzed and compared to healthy controls (HC). Significant differences in the levels of high-mannose and sialylated structures were revealed by the glycan data, which may have important implications for disease pathology. The analysis of serum N-glycosylation profiles indicated significant distinctions between Parkinson’s disease (PD) patients and healthy controls, particularly regarding high-mannose and sialylated glycan structures. Table 1 summarizes the mean percentages and standard deviations of various glycan structures analyzed in the study. Key findings include that a mean percentage of FA2 was recorded at 3.34% (*p* = 0.28), indicating no significant difference between PD patients and healthy controls, suggesting a baseline glycosylation state.

**Figure 1:**
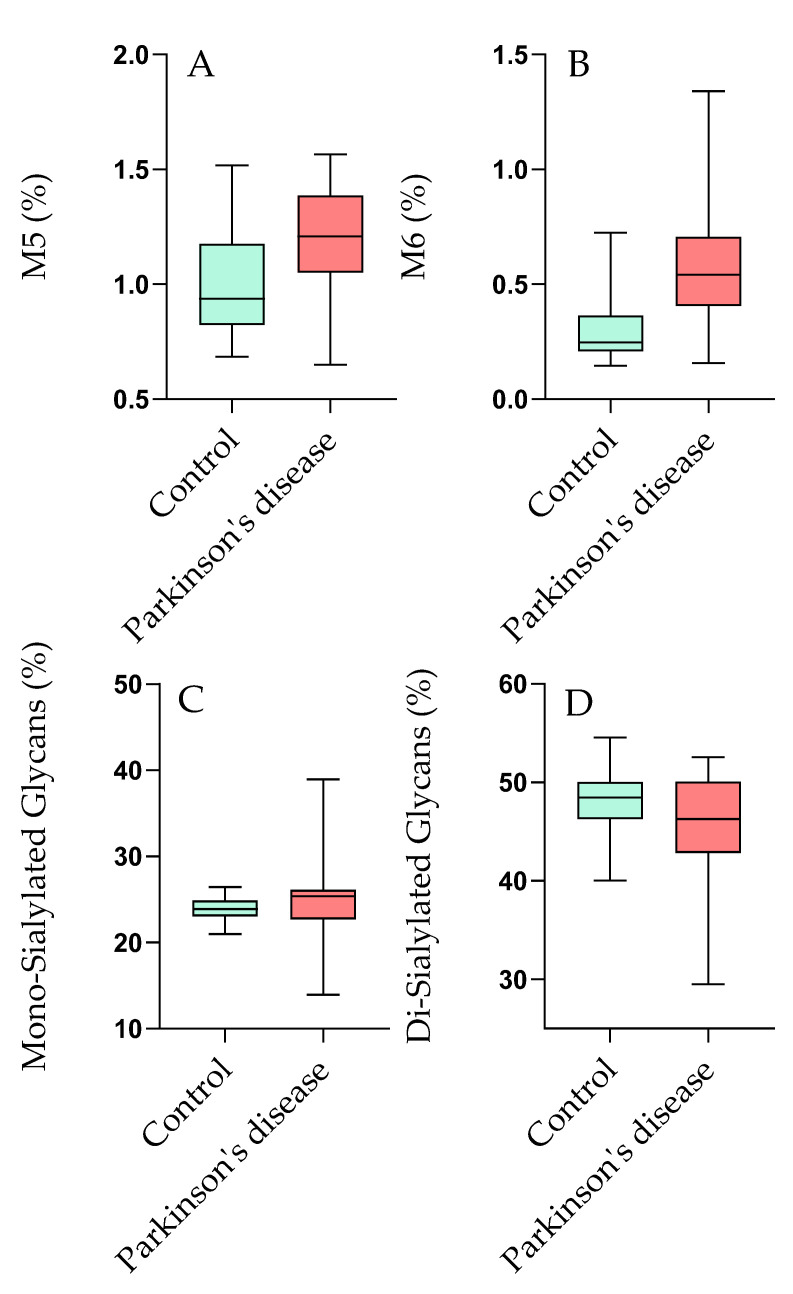
Comparison of Glycan Percentages between Control and Parkinson’s Disease Groups. Box plots illustrating the percentage of (**A**) M5, (**B**) M6, (**C**) Mono-Sialylated Glycans, and (**D**) Di-Sialylated Glycans in individuals diagnosed with Parkinson’s disease (red boxes) compared to healthy controls (green boxes). The y-axis indicates the percentage of each glycan type, while the x-axis shows the two groups being compared. Whiskers represent the range of data, and the boxes indicate the interquartile range, with the line inside each box representing the median.

In contrast, a significant elevation in high-mannose glycans (M5 and M6) was exhibited by PD patients, as shown in Figure 1A,B. Furthermore, the mean level of mono-sialylated glycans (M6) was significantly reduced to 0.47% in PD patients (*p* < 0.001), a finding that correlates with previous studies [19], indicating that diminished sialylation can lead to pro-inflammatory responses and potentially exacerbate neuroinflammation—a critical factor in PD. The results regarding di-sialylated glycan structures also indicated significantly lower levels in PD patients, supporting the conclusions of Paton et al. [20], who highlighted that reduced di-sialylated glycoproteins may correlate with increased neuroinflammatory markers. These findings illustrate a clear relationship between glycan alterations and neurodegenerative processes, emphasizing the importance of glycosylation in the context of PD. Figure 1 visually represents the significant glycosylation-based differences between PD patients and healthy controls as determined by the Kruskal–Wallis test, emphasizing the specific glycan structures that showed statistically significant alterations.

**Figure 2:**
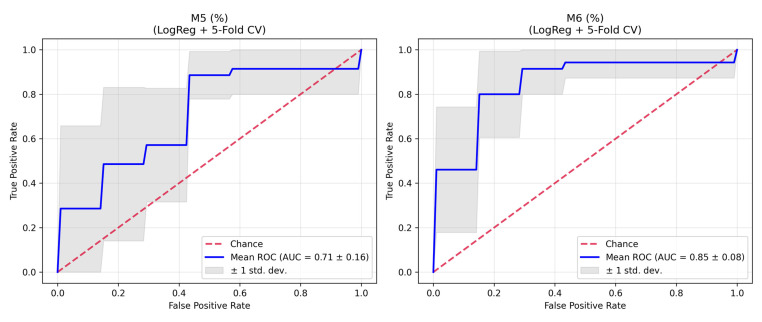
Receiver Operating Characteristic (ROC) curves for glycan profiles M5 (%) and M6 (%) evaluated using Logistic Regression with 5-Fold Cross-Validation. (**Left Panel**) (M5%): Mean Area Under the Curve (AUC) = 0.71 ± 0.16, indicating moderate discriminative ability. (**Right Panel**) (M6%): Mean Area Under the Curve (AUC) = 0.85 ± 0.08, demonstrating strong discriminative ability. In both panels, the blue line represents the mean ROC curve, while the shaded region indicates ±1 standard deviation. The red dashed line represents the chance level (AUC = 0.5). The curves illustrate the trade-off between the true positive rate and false positive rate for each glycan profile.

To ensure statistical robustness and address the issue of multiple comparisons inherent in glycomics data, the Benjamini–Hochberg False Discovery Rate (FDR) correction was applied to all statistical tests (Supplementary Table S1). In the comparison across all four study groups (Control Male, Control Female, Parkinson Male, Parkinson Female) using the Kruskal–Wallis test, statistically significant differences were retained after FDR correction for M6 (FDR-adjusted *p* = 0.0073), M5 (FDR-adjusted *p* = 0.0190), A2BG1S1 (FDR-adjusted *p* = 0.0190), and FA2(6)G1 (FDR-adjusted *p* = 0.0389). Pairwise comparisons using the Mann–Whitney U test revealed that these alterations were driven primarily by the male cohort. In the total PD vs. Control comparison, M6 showed highly significant elevation (FDR-adjusted *p* = 0.00018), followed by A2BG1S1 (FDR-adjusted *p* = 0.0062) and M5 (FDR-adjusted *p* = 0.0296). When stratified by sex, the male cohort exhibited significant differences in M6 (FDR-adjusted *p* = 0.0005), M5 (FDR-adjusted *p* = 0.0336), and A2BG1S1 (FDR-adjusted *p* = 0.0336). In contrast, in the female cohort, no glycans retained statistical significance after FDR correction (e.g., M6: FDR-adjusted *p* = 0.235; M5: FDR-adjusted *p* = 0.895), indicating that the observed glycomic alterations are sex-specific. To evaluate the diagnostic performance of the high-mannose glycans (M5 and M6) while mitigating the risk of overfitting, we performed a Stratified 5-Fold Cross-Validation (CV) with logistic regression. M6 (%) demonstrated robust diagnostic potential with a mean AUC of 0.85 (SD: 0.08) and a bootstrap-validated 95% Confidence Interval (CI) of 0.73–0.93. M5 (%) showed a mean AUC of 0.71 (SD: 0.16) with a 95% CI of 0.61–0.85 (Supplementary Table S2). These validated ROC results confirm that the discriminatory power of M6, and to a lesser extent M5, is stable and reproducible within this cohort, particularly for male patients (Figure 2).

**Figure 3:**
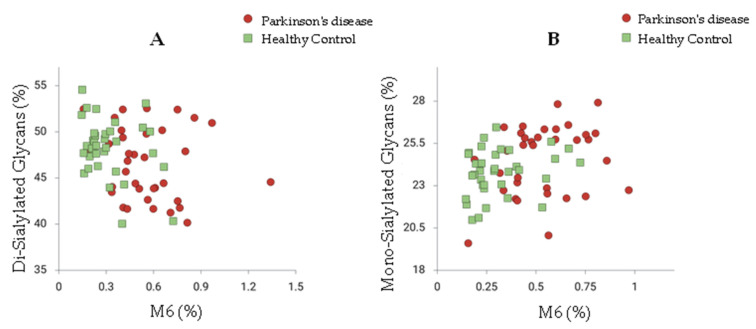
Comparison of Di-Sialylated and Mono-Sialylated Glycans in Parkinson’s Disease and Healthy Controls. Scatter plots displaying the percentage of (**A**) Di-Sialylated Glycans and (**B**) Mono-Sialylated Glycans in individuals diagnosed with Parkinson’s disease (red circles) compared to healthy controls (green squares). Data points represent individual measurements, with overlapping points shown with reduced opacity to enhance visibility. The x-axis represents the percentage of M6, while the y-axis indicates the percentage of glycan types.

Figure 3 presents a scatter plot that compares the glycan ratios of Parkinson’s disease (PD) patients and healthy controls (HC), specifically focusing on the M6/Di-sialylated glycan ratio and the M6/Mono-sialylated glycan ratio. The plot illustrates a noticeable slight separation between the two groups, indicating distinct glycan profiles associated with PD. This separation suggests that the ratios of these glycan structures may serve as potential features for differentiating PD patients from healthy individuals. The findings underscore the role of glycan alterations in the pathophysiology of Parkinson’s disease and support the idea that specific glycan profiles could be utilized for early diagnosis and monitoring of disease progression. These results underscore the relevance of glycosylation changes in PD pathology and their potential clinical implications. Furthermore, the sex-specific differences observed in glycosylation patterns in this study echo prior research, suggesting that hormonal influences may modulate glycosylation and affect disease outcomes and treatment responses. This complexity highlights the necessity of considering sex as a biological variable in PD research. In summary, the significant alterations in serum N-glycosylation patterns between PD patients and healthy controls reveal critical insights into the disease mechanisms and highlight the potential of glycan profiling as a transformative approach in PD management. Future research focusing on the mechanistic underpinnings of these glycan changes and their relationship with disease progression will be essential for advancing the understanding of Parkinson’s disease and improving patient outcomes.

### 2.2. Sex-Associated Differences in Parkinson’s Male Patients

**Figure 4:**
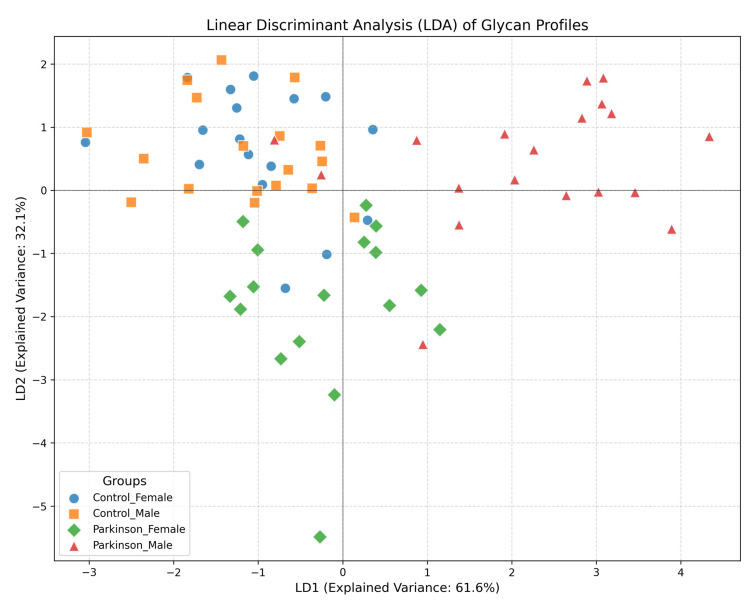
Linear Discriminant Analysis (LDA) of Glycan Profiles. Scatter plot depicting the results of a Linear Discriminant Analysis (LDA) on glycan profiles, illustrating the separation between different groups. The x-axis represents the first linear discriminant (LD1), which explains 61.6% of the variance, while the y-axis represents the second linear discriminant (LD2), explaining 23.1% of the variance. Groups are indicated as follows: Control_Female: Blue circles, Control_Male: Orange squares, Parkinson_Female: Red triangles, Parkinson_Male: Green diamonds. Data points represent individual samples, highlighting the clustering tendencies among the groups.

**Figure 5:**
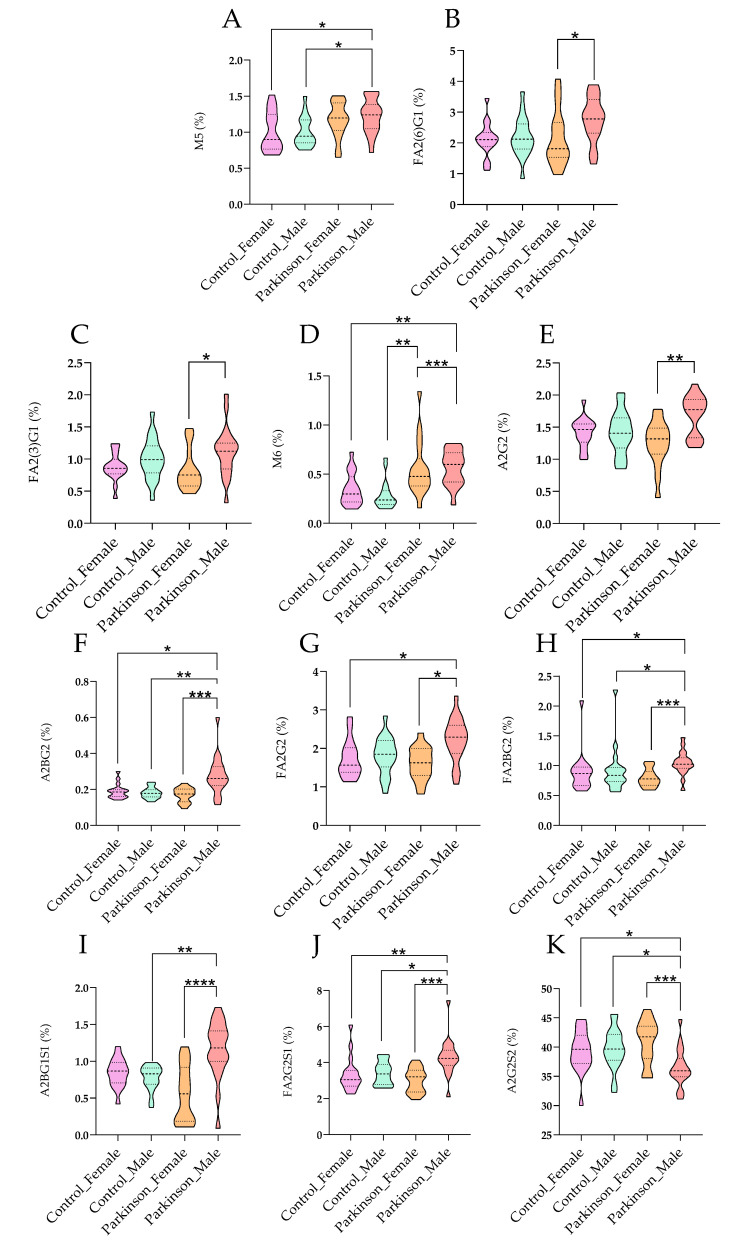
Comparison of Glycan Percentages among Different Groups. Violin plots displaying the percentage of various glycan types across four groups: Control Female, Control Male, Parkinson Female, and Parkinson Male. Each panel illustrates a different glycan or measurement: (**A**) M5 (%), (**B**) FA2(6)G1 (%), (**C**) FA2(3)G1 (%), (**D**) M6 (%), (**E**) A2G2 (%), (**F**) A2BG2 (%), (**G**) FA2G2 (%), (**H**) FA2BG2 (%), (**I**) A2BG1S1 (%), (**J**) FA2G2S1 (%), (**K**) A2G2S2 (%). Statistical significance is indicated by asterisks: * *p* < 0.05, ** *p* < 0.01, *** *p* < 0.001, **** *p* < 0.0001. Whiskers represent the interquartile range, and the median is indicated within each violin.

Figure 4 presents compelling evidence of significant sex-associated differences in glycosylation patterns observed in male patients diagnosed with Parkinson’s disease. This figure emphasizes the unique alterations in serum N-glycosylation profiles that are exhibited by male patients compared to female patients and healthy controls, underscoring the critical importance of considering sex as a biological variable in Parkinson’s disease research. The results indicate that distinct modifications in specific glycan structures are shown by male patients with PD, particularly high-mannose and sialylated glycans. It is suggested that these changes may reflect underlying biological mechanisms influenced by sex, potentially impacting disease progression and therapeutic outcomes. Previous studies, suggest that hormonal influences can modulate glycosylation patterns, which may contribute to variations in disease severity and treatment responses between sexes [8]. This aligns with the concept that sex-specific biological factors can influence neurodegenerative disease mechanisms, a notion supported by emerging literature highlighting the role of sex hormones in neuroinflammatory processes. The alterations in glycosylation observed in male patients have significant implications for understanding the pathophysiology of Parkinson’s disease. Glycosylation is recognized as playing a vital role in the stability and functionality of glycoproteins, including alpha-synuclein, which is known for its pathological aggregation in PD. Studies have shown that aberrant glycosylation can affect protein folding and aggregation propensity, which are critical processes in neurodegenerative conditions [10]. The findings in Figure 4 suggest that glycan changes in male patients may exacerbate neuroinflammatory responses, thereby contributing to the progression of the disease. The sex-specific glycosylation alterations highlighted in Figure 4 could pave the way for developing novel strategies that enhance diagnostic accuracy in Parkinson’s disease. In conclusion, Figure 4 serves as a crucial visual representation of the significant sex-associated differences in glycosylation patterns among male PD patients. It not only enhances the understanding of the complex mechanisms underlying Parkinson’s disease but also reflects the growing body of literature that emphasizes the necessity of examining sex differences in neurodegenerative disease research. These insights highlight the potential for glycan profiling to inform clinical practices, ultimately improving patient outcomes through more personalized approaches to diagnosis and treatment. Figure 5 presents the results of a Linear Discriminant Analysis (LDA) based on serum N-glycan profiles from Parkinson’s disease (PD) patients and healthy controls (HC), analyzed separately by sex. Each point represents an individual glycan pattern, while the colored ellipses indicate 95% confidence intervals for the four subgroups (Parkinson_Male, Parkinson_Female, Control_Male, Control_Female) along the first two discriminant axes.

The LDA model demonstrates a clear separation between PD and control groups, most prominently among male patients, whose glycan profiles cluster distinctly along the first discriminant axis. This separation reflects the presence of disease-specific glycosylation signatures, which appear to be more pronounced in males. In contrast, female PD samples show partial overlap with female controls, indicating sex-dependent variability in the extent and direction of glycan alterations associated with Parkinson’s disease. The partial overlap observed between male and female control groups likely represents physiological glycan variability, potentially influenced by hormonal and metabolic regulation. Overall, the LDA results reveal distinct sex- and disease-specific segregation of serum N-glycan patterns, supporting the hypothesis that altered glycosylation constitutes an early and sensitive attribute of Parkinson’s disease pathophysiology. These findings highlight the importance of considering sex as a biological variable in glycomics research, particularly in the context of neurodegenerative diseases like Parkinson’s. The pronounced glycan alterations in males could suggest a differential pathway activation or hormonal influence that warrants further investigation. Additionally, the overlapping patterns in females suggest a more complex interplay of factors that may modulate glycosylation changes, possibly including estrogen levels and other sex-specific physiological processes. Future studies should aim to dissect these sex-specific differences further, potentially incorporating other omics data to unravel the underlying mechanisms. Understanding the precise role of glycosylation in Parkinson’s disease could pave the way for the development of sex-specific diagnostic tools and therapeutic strategies, enhancing personalized medicine approaches for this condition. Moreover, longitudinal studies are needed to determine whether these glycan alterations precede clinical symptoms and could be utilized for early detection or monitoring disease progression.

## 3. Discussion

The glycosylation patterns identified in this study reveal significant differences between Parkinson’s disease (PD) patients and healthy controls, particularly in the levels of high-mannose and sialylated glycans. The increased prevalence of high-mannose glycans in PD patients suggests alterations in glycan biosynthesis, which may influence protein folding and aggregation. The results are consistent with previous studies that have identified similar glycosylation changes in other neurodegenerative conditions. For instance, it was demonstrated that altered glycosylation patterns in Alzheimer’s disease correlate with disease progression, suggesting that glycosylation may serve as a common pathological feature across neurodegenerative disorders [21]. The sex-specific differences observed in this study further highlight the complexity of PD, as it is suggested that hormonal influences may modulate glycosylation patterns, potentially affecting disease outcomes and treatment responses [22]. The implications of these findings extend to clinical practice, particularly regarding the potential for using glycan patterns for early diagnosis of PD. The ROC analysis showing AUC values of 0.71 for M5 and 0.85 for M6 indicates that these glycan changes could facilitate earlier therapeutic interventions, ultimately improving patient outcomes. The increased levels of high-mannose glycans, particularly M5 and M6, observed in our study may be indicative of underlying cellular stress responses rather than solely reflecting disease-related biosynthetic alterations. High-mannose glycan accumulation can arise from several physiological and pathological processes, particularly those associated with endoplasmic reticulum (ER) stress and protein quality control mechanisms. Furthermore, it has been suggested that targeting glycosylation pathways could provide novel therapeutic strategies, as manipulating glycan structures may alter the aggregation behavior of proteins involved in PD. Figure 4 illustrates pronounced differences in glycan signatures between male and female patients diagnosed with Parkinson’s disease (PD). These distinctions may be attributed to several factors, including hormonal influences, genetic variability, and differences in immune response between sexes. In contrast, the glycan profiles of healthy male and female controls show less variation. This could suggest that the metabolic and physiological changes associated with PD exacerbate sex-specific glycosylation patterns, revealing underlying biological mechanisms influenced by the disease’s progression. Moreover, the altered glycosylation may reflect the distinct pathophysiological processes occurring in male and female PD patients, potentially related to differences in disease severity, progression rates, or responses to environmental factors. Understanding these differences could provide insights into the role of sex as a biological variable in neurodegenerative diseases, guiding personalized therapeutic approaches in the future.

Despite the promising insights provided by our study into the glycosylation patterns in Parkinson’s disease (PD), several limitations must be acknowledged. The relatively small sample size, consisting of only 35 PD patients and 35 healthy controls, restricts the statistical power and generalizability of our findings. A larger cohort would facilitate more robust conclusions and allow for detailed subgroup analyses. Additionally, the presence of diverse comorbidities among participants complicates the interpretation of the results, as it is common for older individuals to present with multiple health conditions that could confound the relationship between glycosylation and PD. This variability makes it challenging to isolate the specific impacts of PD on glycosylation patterns. Furthermore, while critical clinical variables such as disease duration and dopaminergic medication status are detailed in the included table, their absence as controlled factors in our analyses limits the clarity of our findings. Acknowledging these limitations is essential for understanding the context of our results and highlights the need for future research with larger, more homogeneous cohorts.

## 4. Materials and Methods

### 4.1. Chemicals

Formic acid, ammonium hydroxide, acetic acid, acetonitrile, picoline borane, procainamide hydrochloride, and dimethyl sulfoxide were obtained from Sigma-Aldrich (St. Louis, MO, USA). PNGase F was acquired from New England Biolabs (Ipswich, MA, USA).

### 4.2. Patient Samples

Serum samples were collected from 70 patients categorized into 2 groups: 35 healthy controls (average age 64.18 ± 8.62) and 35 diagnosed with Parkinson’s disease (average age 69.46 ± 6.34) with 18 females and 17 males in both groups. The samples were obtained at Borsod Academic County Hospital (Miskolc, Hungary). Blood samples were collected using sterile vacutainer tubes without anticoagulants. After collection, the samples were allowed to clot at room temperature for approximately 30 min, facilitating the separation of serum from cellular components. Subsequently, samples were centrifuged at 2000 RPM for 10–15 min. The serum was carefully pipetted into new sterile tubes, ensuring that the clot remained undisturbed. For storage, samples were frozen at −80 °C, depending on the requirements of the intended analysis. Supplementary Table S3 summarizes the baseline characteristics of the patient samples. This study received approval from the Regional Research Ethics Committee (ethical approval number: BORS-19/2023), and written informed consent was provided by all patients in accordance with the Declaration of Helsinki.

### 4.3. N-Glycan Release from Serum Proteins, Labeling, and Clean-Up

N-glycan release was conducted following the PNGase F deglycosylation protocol from New England Biolabs (Ipswich, MA, USA) using 9 µL of serum sample. The released carbohydrates were fluorescently labeled by adding 10 μL of a 0.3 M procainamide and 300 mM picoline borane solution in a 70%/30% dimethyl sulfoxide/acetic acid mixture, followed by incubation at 65 °C for 4 h. The labeled glycans were then purified using NH_2_-functionalized MonoSpin columns (GL Sciences Inc., Tokyo, Japan) according to the manufacturer’s instructions. The purified glycans were dissolved in a 25%/75% water/acetonitrile solution and analyzed using HILIC-UPLC-FLR-MS.

### 4.4. UPLC-FLR-MS Analysis

Fluorescently labeled N-glycans were analyzed using a Waters Acquity ultra-performance liquid chromatography (UPLC) system equipped with a fluorescence detector and a Xevo-G2S qTOF mass spectrometer controlled by MassLynx 4.2 software (Waters, Milford, MA, USA). Separation was performed on a Waters BEH Glycan column (100 × 2.1 mm i.d., 1.7 μm particle size) with a linear gradient of 75–55% acetonitrile (Buffer B) at a flow rate of 0.4 mL/min over 22 min, using 50 mM ammonium formate (pH 4.4) as Buffer A. Each run used a 1 μL injection volume, with the sample manager set at 15 °C and the column maintained at 60 °C. Fluorescence detection was performed with excitation and emission wavelengths of λex = 308 nm and λem = 359 nm, respectively. Mass spectrometry (MS) analysis was conducted in positive ionization mode, applying a 3 kV electrospray voltage to the capillary. The desolvation temperature was set to 120 °C, with a desolvation gas flow rate of 800 L/h. Mass spectra were acquired over the 500–3000 *m*/*z* range.

### 4.5. Data Analysis

Chromatograms of the patient samples were integrated using Unifi chromatography software 1.6 (Waters, Milford, MA, USA) based on fluorescence spectra, with MS confirmation. The mass-to-charge ratios of individual glycan structures were determined using GlycoWorkbench 2.0. Statistical analyses, including the Kruskal–Wallis test and Mann–Whitney pairwise comparisons, were performed using IBM SPSS Statistics 23. Linear discriminant analysis was conducted with Past 4.11 software. Figures were generated using GraphPad Prism 10.

Normalization was not required for our analysis, as relative area percentages of the chromatographic peaks were used. This method accounts for variations in sample concentration and facilitates direct comparison of glycan profiles across samples. By focusing on the relative areas, we ensured that the reported glycan differences accurately represent the underlying biological variations among the examined samples.

Statistical analyses were performed using Python (version 3.x), utilizing the pandas, scipy, statsmodels, and scikit-learn libraries. Given the non-normal distribution of the data, non-parametric tests were employed. Differences between the four study groups (Control Male, Control Female, Parkinson Male, Parkinson Female) were evaluated using the Kruskal–Wallis test. Pairwise comparisons were conducted using the Mann–Whitney U test. To control for the false discovery rate (FDR) due to multiple testing, the Benjamini–Hochberg procedure was applied to all *p*-values; an FDR-adjusted *p*-value (q-value) of <0.05 was considered statistically significant.

To assess the diagnostic potential of specific glycans (M5 and M6) and address concerns regarding overfitting in this exploratory cohort, we performed Receiver Operating Characteristic (ROC) curve analysis with internal validation. A Logistic Regression model was employed within a Stratified 5-Fold Cross-Validation framework. This approach ensured that the Area Under the Curve (AUC) estimates were robust and not artifacts of the specific data split. Furthermore, 95% confidence intervals (CI) for the AUC values were calculated using bootstrapping with 1000 iterations.

Additionally, Linear Discriminant Analysis (LDA) was conducted as a supervised dimensionality reduction technique to visualize the global separation of glycan profiles between the study groups and to investigate the multivariate relationship of the measured glycans.

## 5. Conclusions

In summary, this study reveals significant alterations in serum N-glycosylation patterns between male Parkinson’s disease (PD) patients and healthy controls, underscoring the importance of considering sex as a biological variable in neurodegenerative research. The findings indicate that increased levels of high-mannose glycans and reduced mono-sialylated glycans in male patients might be helpful for distinguishing PD from healthy states. While the ROC analysis demonstrates encouraging AUC values of 0.733 for M5 and 0.837 for M6, further validation through independent cohorts is essential to confirm these findings and mitigate concerns regarding overfitting. The sex-specific differences observed warrant a cautious interpretation, as they highlight the complexity of glycosylation alterations influenced by hormonal and biological factors. This research not only enhances our understanding of the pathophysiological mechanisms underlying Parkinson’s disease but also points towards the potential utility of glycan profiling in early diagnosis and monitoring. Future studies should focus on longitudinal analyses and mechanistic investigations to further elucidate the relationship between glycosylation changes and disease progression, thereby paving the way for innovative diagnostic and therapeutic strategies tailored to male patients with Parkinson’s disease.

## Figures and Tables

**Table 1:** Summary of glycan structures with their mean percentages, standard deviations, and significance levels. The table displays various glycan types, including FA2, M5, and A2G2, highlighting their respective values in control and Parkinson’s disease cohorts.

Structures	Mean	Std. Deviation	Sig.
FA2 (%)	3.34	1.46	0.28
M5 (%)	1.16	0.57	0.00
FA2B (%)	1.21	0.47	1.00
FA2(6)G1 (%)	2.31	0.76	0.02
FA2(3)G1 (%)	0.96	0.33	0.03
FA2BG1 (%)	1.16	0.35	0.39
M6 (%)	0.47	0.34	0.00
A2G2 (%)	1.49	0.54	0.01
A2BG2 (%)	0.21	0.08	0.00
FA2G2 (%)	1.86	0.54	0.01
FA2BG2 (%)	0.96	0.47	0.00
A2BG1S1 (%)	0.84	0.37	0.00
A2G2S1 (%)	13.85	1.63	0.52
FA2G2S1 (%)	3.54	0.96	0.00
FA2BG2S1 (%)	6.01	2.11	0.80
A2G2S2 (%)	38.75	4.54	0.00
FA2G2S2 (%)	3.93	0.98	0.65
FA2BG2S2 (%)	2.68	1.29	0.44
A2BG2S2 (%)	1.79	0.42	0.93
A3G3S2 (%)	1.41	0.64	0.95
A3G3S3 (%)	5.55	1.55	0.25
FA3G3S3 (%)	5.62	1.61	0.61
A4G4S3 (%)	0.54	0.20	0.31
A4G4S4 (%)	0.37	0.16	0.43
Non-Sialylated Glycans (%)	13.50	3.37	0.01
Mono-Sialylated Glycans (%)	24.23	2.77	0.00
Di-Sialylated Glycans (%)	47.15	4.16	0.00
De-Fucosylated Glycans (%)	56.08	5.26	0.02
Antennary 2 Glycans (%)	84.89	1.88	0.62

## Data Availability

The original contributions presented in this study are included in the article/Supplementary Material. Further inquiries can be directed to the corresponding author(s).

## Supplementary Materials

The following supporting information can be downloaded at: https://www.mdpi.com/article/10.3390/ijms27010552/s1.
