# Supplementary material for: Differential Glycosylation Patterns in Parkinson’s Disease: Emphasis on Male-Specific Changes Identified via HILIC-LC-MS
Source: Int J Mol Sci. 2026 Jan 5;27(1):552. doi: 10.3390/ijms27010552 (PMC12787113; doi:10.3390/ijms27010552)
Supplement: Supplementary file 1 [file ijms-27-00552-s001.zip › ijms-4063173-supplementary.pdf]

International Journal of *Molecular Sciences*

MDPI

Supplementary Material

Differential Glycosylation Patterns in Parkinson's Disease: Emphasis on Male-Specific Changes Identified via HILIC-LC-MS

Béla Demeter [1,2], Adriána Kutás [1], Béla Viskolcz [1], Csaba Oláh [2], Edina Petercsák [2], Attila Garami [3] and Csaba Váradi [1,*]

[1] Institute of Chemistry, Faculty of Materials Science and Chemical Engineering, University of Miskolc, 3515 Miskolc, Hungary; demeter.bela@gmail.com (B.D.); kutas.adriana@student.uni-miskolc.hu (A.K.); bela.viskolcz@uni-miskolc.hu (B.V.)
[2] Borsod-Abaúj-Zemplén County Center Hospital and University Teaching Hospital, Department of Neurosurgery, 3526 Miskolc, Hungary; olahcs@gmail.com (C.O.); petercsakedina@gmail.com (E.P.)
[3] Institute of Energy, Ceramic and Polymer Technology, University of Miskolc, 3515 Miskolc, Hungary; attila.garami@uni-miskolc.hu
* Correspondence: csaba.varadi@uni-miskolc.hu; Tel.: +30-894-7730

Academic Editor: Cosima Damiana Calvano

Received: 9 December 2025
Revised: 31 December 2025
Accepted: 1 January 2026
Published: 5 January 2026

Copyright: © 2026 by the authors. Licensee MDPI, Basel, Switzerland. This article is an open access article distributed under the terms and conditions of the Creative Commons Attribution (CC BY) license.

Table S1. Statistical analysis of glycan profiles in Parkinson's disease patients compared to control groups using the Kruskal-Wallis test. The table presents p-values for each glycan, indicating the significance of differences across four groups and between Parkinson's disease and control subjects (both overall and stratified by gender). Adjusted p-values using the False Discovery Rate (FDR) are also included, highlighting the glycan markers that show significant differences in their distributions.

Glycan	Test	p-value	p-value (FDR)
FA2 (%)	Kruskal-Wallis (4 Groups)	0,279148286	0,515350682
M5 (%)	Kruskal-Wallis (4 Groups)	0,007132469	0,019019919
FA2B (%)	Kruskal-Wallis (4 Groups)	0,997769575	0,997769575
FA2(6)G1 (%)	Kruskal-Wallis (4 Groups)	0,016198811	0,038877147
FA2(3)G1 (%)	Kruskal-Wallis (4 Groups)	0,029986938	0,065426047
FA2BG1 (%)	Kruskal-Wallis (4 Groups)	0,387996591	0,617204274
M6 (%)	Kruskal-Wallis (4 Groups)	1,22199E-05	0,000293277
A2G2 (%)	Kruskal-Wallis (4 Groups)	0,004652583	0,014133121
A2BG2 (%)	Kruskal-Wallis (4 Groups)	0,000243389	0,001460337
FA2G2 (%)	Kruskal-Wallis (4 Groups)	0,00471104	0,014133121
FA2BG2 (%)	Kruskal-Wallis (4 Groups)	0,001141104	0,005472235
A2BG1S1 (%)	Kruskal-Wallis (4 Groups)	4,47693E-05	0,000537231
A2G2S1 (%)	Kruskal-Wallis (4 Groups)	0,517861803	0,690482404
FA2G2S1 (%)	Kruskal-Wallis (4 Groups)	0,000165039	0,001320308
FA2BG2S1 (%)	Kruskal-Wallis (4 Groups)	0,795306454	0,908921662
A2G2S2 (%)	Kruskal-Wallis (4 Groups)	0,001368059	0,005472235
FA2G2S2 (%)	Kruskal-Wallis (4 Groups)	0,645819961	0,774983953
FA2BG2S2 (%)	Kruskal-Wallis (4 Groups)	0,437186361	0,617204274
A2BG2S2 (%)	Kruskal-Wallis (4 Groups)	0,926670386	0,986090893
A3G3S2 (%)	Kruskal-Wallis (4 Groups)	0,945003773	0,986090893
A3G3S3 (%)	Kruskal-Wallis (4 Groups)	0,250500361	0,501000721
FA3G3S3 (%)	Kruskal-Wallis (4 Groups)	0,613825195	0,774983953

 https://doi.org/10.3390/ijms27010552

A4G4S3 (%)	Kruskal-Wallis (4 Groups)	0,307017635	0,526315945
A4G4S4 (%)	Kruskal-Wallis (4 Groups)	0,427278015	0,617204274
FA2 (%)	PD vs Control (All)	0,894776616	0,933679947
M5 (%)	PD vs Control (All)	0,000768022	0,009216261
FA2B (%)	PD vs Control (All)	0,949562645	0,949562645
FA2(6)G1 (%)	PD vs Control (All)	0,1012329	0,382604995
FA2(3)G1 (%)	PD vs Control (All)	0,867554295	0,933679947
FA2BG1 (%)	PD vs Control (All)	0,238453132	0,440221168
M6 (%)	PD vs Control (All)	1,11196E-06	2,66871E-05
A2G2 (%)	PD vs Control (All)	0,372749648	0,596399438
A2BG2 (%)	PD vs Control (All)	0,045994958	0,367959666
FA2G2 (%)	PD vs Control (All)	0,195709318	0,440221168
FA2BG2 (%)	PD vs Control (All)	0,119138917	0,382604995
A2BG1S1 (%)	PD vs Control (All)	0,233903714	0,440221168
A2G2S1 (%)	PD vs Control (All)	0,207896848	0,440221168
FA2G2S1 (%)	PD vs Control (All)	0,124686618	0,382604995
FA2BG2S1 (%)	PD vs Control (All)	0,58485843	0,738768543
A2G2S2 (%)	PD vs Control (All)	0,152176439	0,405803838
FA2G2S2 (%)	PD vs Control (All)	0,308750815	0,529287111
FA2BG2S2 (%)	PD vs Control (All)	0,127534998	0,382604995
A2BG2S2 (%)	PD vs Control (All)	0,530784083	0,738768543
A3G3S2 (%)	PD vs Control (All)	0,576978918	0,738768543
A3G3S3 (%)	PD vs Control (All)	0,08980804	0,382604995
FA3G3S3 (%)	PD vs Control (All)	0,84949091	0,933679947
A4G4S3 (%)	PD vs Control (All)	0,576978918	0,738768543
A4G4S4 (%)	PD vs Control (All)	0,743077148	0,891692577
FA2 (%)	PD vs Control (Male)	0,218447181	0,339322272
M5 (%)	PD vs Control (Male)	0,002761544	0,011046176
FA2B (%)	PD vs Control (Male)	0,867269564	0,867269564
FA2(6)G1 (%)	PD vs Control (Male)	0,014439415	0,041873653
FA2(3)G1 (%)	PD vs Control (Male)	0,242043642	0,339322272
FA2BG1 (%)	PD vs Control (Male)	0,104014169	0,208028338
M6 (%)	PD vs Control (Male)	4,36927E-05	0,001048624
A2G2 (%)	PD vs Control (Male)	0,025520342	0,061248822
A2BG2 (%)	PD vs Control (Male)	0,000502735	0,004021878
FA2G2 (%)	PD vs Control (Male)	0,01570262	0,041873653
FA2BG2 (%)	PD vs Control (Male)	0,006535626	0,02240786
A2BG1S1 (%)	PD vs Control (Male)	0,000197346	0,002368152
A2G2S1 (%)	PD vs Control (Male)	0,218447181	0,339322272
FA2G2S1 (%)	PD vs Control (Male)	0,001841616	0,011046176
FA2BG2S1 (%)	PD vs Control (Male)	0,553486569	0,642888533
A2G2S2 (%)	PD vs Control (Male)	0,002498755	0,011046176
FA2G2S2 (%)	PD vs Control (Male)	0,230030295	0,339322272
FA2BG2S2 (%)	PD vs Control (Male)	0,475170835	0,600215792
A2BG2S2 (%)	PD vs Control (Male)	0,594885314	0,642888533
A3G3S2 (%)	PD vs Control (Male)	0,616101511	0,642888533
A3G3S3 (%)	PD vs Control (Male)	0,070603707	0,154044452
FA3G3S3 (%)	PD vs Control (Male)	0,594885314	0,642888533
A4G4S3 (%)	PD vs Control (Male)	0,132542907	0,244694597
A4G4S4 (%)	PD vs Control (Male)	0,254491704	0,339322272
FA2 (%)	PD vs Control (Female)	0,35238384	0,866745024

M5 (%)	PD vs Control (Female)	0,091462481	0,716702325
FA2B (%)	PD vs Control (Female)	1	1
FA2(6)G1 (%)	PD vs Control (Female)	0,704777829	0,894737208
FA2(3)G1 (%)	PD vs Control (Female)	0,179175581	0,716702325
FA2BG1 (%)	PD vs Control (Female)	1	1
M6 (%)	PD vs Control (Female)	0,009787015	0,234888349
A2G2 (%)	PD vs Control (Female)	0,157895551	0,716702325
A2BG2 (%)	PD vs Control (Female)	0,469486888	0,866745024
FA2G2 (%)	PD vs Control (Female)	0,654322144	0,894737208
FA2BG2 (%)	PD vs Control (Female)	0,428243348	0,866745024
A2BG1S1 (%)	PD vs Control (Female)	0,027497206	0,329966473
A2G2S1 (%)	PD vs Control (Female)	0,836274467	0,912299419
FA2G2S1 (%)	PD vs Control (Female)	0,469486888	0,866745024
FA2BG2S1 (%)	PD vs Control (Female)	0,730518931	0,894737208
A2G2S2 (%)	PD vs Control (Female)	0,214987544	0,737100152
FA2G2S2 (%)	PD vs Control (Female)	0,756567201	0,894737208
FA2BG2S2 (%)	PD vs Control (Female)	0,179175581	0,716702325
A2BG2S2 (%)	PD vs Control (Female)	0,581567517	0,894737208
A3G3S2 (%)	PD vs Control (Female)	0,782895057	0,894737208
A3G3S3 (%)	PD vs Control (Female)	0,581567517	0,894737208
FA3G3S3 (%)	PD vs Control (Female)	0,317861339	0,866745024
A4G4S3 (%)	PD vs Control (Female)	0,469486888	0,866745024
A4G4S4 (%)	PD vs Control (Female)	0,654322144	0,894737208

Table S2. Area Under the Curve (AUC) values for glycan profiles M5 and M6 as potential biomarkers in Parkinson's disease. The table displays the mean AUC, standard deviation (Std), and 95% confidence intervals (CI) for both M5 (%) and M6 (%). These values indicate the diagnostic performance of each biomarker, with M6 showing a higher AUC, suggesting better discrimination between disease and control groups.

Biomarker	AUC (Mean)	AUC (Std)	95% CI Lower	95% CI Upper
M5 (%)	0,710204082	0,15	0,60	0,84
M6 (%)	0,849489796	0,08	0,73	0,93

Table S3. Patient demographics and clinical characteristics. This table summarizes the sample number, disease duration, medications, and comorbidities for each patient, providing insight into the clinical profiles of individuals included in the study.

Sample number	Disease Duration (years)	Medication	Comorbidities
P1	5	Madopar (levodopa, benserazide)	na
P2	11	Akyneton (biperiden)	DM
P4	7	Stalevo (levodopa, carbidopa, entacapone)	Hypecholest, D vit deff.
P6	4	Xadago (safinamide)	Hypercholest. D vit deff.
P7	7	Ralago (rasagiline)	na
P10	2	na	na
P12	5	Oprymea (pramipexole)	Struma, DM

https://doi.org/10.3390/ijms27010552

P14	9	na	na
P15	6	na	na
P16	3	na	DM, Stroke
P17	3	na	DM
P18	5	na	na
P19	7	na	DM, ISZB
P22	2	na	Hypertonia
P25	8	na	Hypertonia
P27	2	na	Hypertonia
P28	5	na	DM
P29	1	na	DM
P31	1	na	hypertonia, struma
P32	2	na	DM, Hypertonia, ISZB
P36	1	na	hypertonia, ISZB, hyperlipid., thyreoditis
P38	1	na	na
P39	1	na	Stroke
P41	2	na	Stroke
P42	2	na	DM
P43	1	na	hypertonia, struma
P44	8	na	hypertonia, ISZB
P47	6	na	na
P51	2	na	hypertonia, ISZB, epilepsia
P53	2	na	Deg. Gerinc
P54	2	na	hypertonia
P55	1	na	Hypertonia
P56	6	na	hypertonia, Crohn
P60	9	na	hypertonia, hyperlipidaemia

Disclaimer/Publisher's Note: The statements, opinions and data contained in all publications are solely those of the individual author(s) and contributor(s) and not of MDPI and/or the editor(s). MDPI and/or the editor(s) disclaim responsibility for any injury to people or property resulting from any ideas, methods, instructions or products referred to in the content.

https://doi.org/10.3390/ijms27010552
